# *In Situ* Multiscale Investigation of Capillary-Force-Induced Cold-Welding of Silver Nanowire Networks

**DOI:** 10.1021/acsomega.5c07063

**Published:** 2025-11-14

**Authors:** Yevheniia Chernukha, Laetitia Bardet, Maxime Berthe, Thomas Lerond, Jean-Paul Mazellier, Laurent Gangloff, Aurore Denneulin, Pascale Diener, Daniel Bellet

**Affiliations:** † Univ. Lille, CNRS, Centrale Lille, Univ. Polytechnique Hauts-de-France, Junia-ISEN, UMR 8520 - IEMN, F-59000 Lille, France; ‡ Univ. Grenoble Alpes, CNRS, Grenoble INP, LMGP, 38000 Grenoble, France; § 84360 Thales Research and Technology, Route Départementale 128, 91767 Palaiseau Cedex, France

## Abstract

Silver nanowire (AgNW) networks are in the spotlight as flexible transparent electrodes (TEs) thanks to their combination of high optical transmittance, low electrical resistance, and excellent flexibility. As such network is formed from a multitude of interwoven AgNW, a postdeposition treatment is needed to get the best electrical conductivity from each interconnect. Traditionally, thermal annealing above 200 °C enhances AgNW junction contacts but restricts compatibility with heat-sensitive substrates such as polymers and perovskites, common in flexible electronics and solar cells. The present study explores the potential of *capillary-force-induced cold-welding*, an already reported low-temperature alternative operating at or below 100 °C. Better insight into the morphological and electrical properties of cold-welded AgNWs is essential for their large-scale integration as flexible and stable TEs in devices. In this work, the effective welding of AgNW junctions is directly demonstrated by *in situ* and nanoscale transport measurements. The junction resistance is drastically reduced while preserving both optical transparency and nanowire morphology at significantly lower temperatures than those used with conventional treatments such as thermal annealing. These results strengthen cold welding as a promising approach for overcoming the challenges of AgNW network integration in flexible electronics.

## Graphical abstract

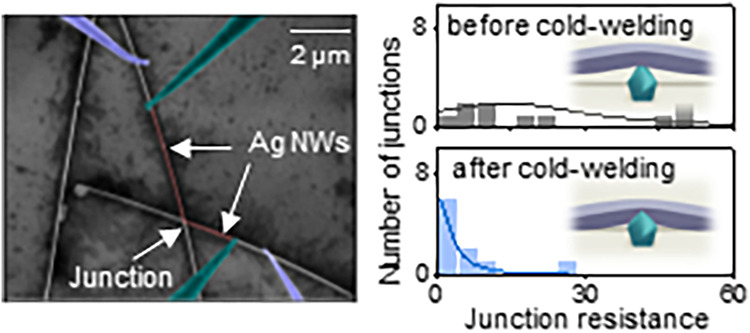

## I Introduction

The growing demand for low-cost and flexible devices and the critical scarcity of indium limit the use of conventional transparent electrodes (TEs) made of sputtered indium tin oxide (ITO).^1^ Silver nanowire (AgNW) networks offer a promising alternative for next-generation transparent conductive materials thanks to their optical transparency, electrical conductivity, and mechanical flexibility.^2,3^ Besides their physical qualities, AgNWs are compatible with suspension-based deposition methods like spray-coating, enabling large-scale production, including roll-to-roll processes^4,5^ commonly used in TE manufacturing.

However, these networks can exhibit high electrical resistance after deposition because of insufficient contact between nanowires, the presence of organic residues, or polyvinylpyrrolidone (PVP) used during the synthesis of AgNWs.^6^ Several routes have been explored that effectively reduce the electrical resistance of metallic nanowire networks, such as the integration of AgNWs with other conductive materials,^7^ and postdeposition treatments,^8,9^ such as thermal annealing,^10–12^ irradiation,^13^ mechanical pressing,^14,15^ capillary-force-induced cold-welding,^16–20^ or chemical treatment.^21,22^ Among these, thermal annealing is the most studied and widely applied method, achieving significant resistance decrease in AgNW networks depending on nanowire dimensions, network density, and surface chemistry.^12,23^ However, thermal annealing requires high temperatures (150–270 °C) and precise temperature control to avoid damage to the morphology of the AgNW.^6^ Because of this limitation, thermal annealing is incompatible with polymer substrates commonly used in flexible TEs or with temperature-sensitive layers such as perovskite tandem silicon solar cells. In this context, capillary-force-induced cold-welding is an excellent low-temperature alternative^17^ and is already in use in preindustrial TE production.^24^ In this process, moisture is applied to the AgNW network at temperatures at or below 100 °C. As the solvent evaporates, liquid menisci form and exert high local pressures between the nanowires and the substrate, especially at the nanowire junctions. These local pressures are expected to effectively weld AgNW within the network and also enhance its adhesion to the substrate.

The aim of any of these post-treatments mainly concerns the optimization of the contact resistances between nanowires, making crucial the evaluation of the electrical properties at the scale of the individual nanowire. A few works successfully assess the electrical resistance at single nanowire–nanowire-junctions, either by modeling large-scale measurements,^25,26^ by direct measurement with lithographed electrodes,^27,28^ or by direct measurement with ultrasharp electrical probes.^29^ The latter method has the advantage of directly probing the single nanowire–nanowire junctions in random configuration without any additional processing, thus with no additional contamination. Using this method, Selzer et al.^29^ compared the electrical resistance at NW–NW junctions in as-deposited and thermally annealed AgNW networks, showing that thermal annealing effectively eliminates the junction resistance and successfully welds the AgNWs.

For AgNWs optimized by cold-welding treatment, the transport properties at the scale of individual nanowires have not yet been reported; thus, investigating and optimizing junction resistance would be of significant interest, with open questions such as the effect of moisture on the AgNW resistivity and possible residual resistances at the NW–NW junction due to the PVP layer. This article presents a comparative study of three types of AgNW networks composed of: (i) as-deposited AgNWs (AD-AgNWs), (ii) thermally annealed AgNWs (TA-AgNWs), and (iii) cold-welded AgNWs (CW-AgNWs). The optimization of the cold-welding process is presented through *in situ* monitoring of the AgNW network resistance during the post-treatment. The optical properties and thermal stability of the three types of networks are compared, as well as the electrical resistance of individual junctions, showing equivalent performances. The electrical resistance of an individual NW–NW junction for TA-AgNWs and CW-AgNWs (*R*_J_^TA^ and *R*_J_^CW^, respectively) is strongly reduced compared to that of AD-AgNWs (*R*_J_^AD^), with a decrease of the mean value from *R*_J_^AD^ = 21 ± 6 Ω to *R*_J_^TA^ = 6 ± 3 Ω and *R*_J_^CW^ = 5 ± 3 Ω. Additionally, the sharp dispersion of the NW–NW junction resistance values of CW-AgNWs and TA-AgNWs evidences a complete welding for most of the NW–NW junctions. Moreover, the resistivities along a single AgNW are similar in the three samples, showing that the two post-treatments do not modify the AgNW structural and electrical properties. These results demonstrate the effectiveness of cold-welding for optimizing transparent AgNW electrodes and the high potential of this method in the preparation of large-scale conducting transparent nanowire networks.

## II Materials and Methods

### II.I Fabrication of AgNW Networks

AgNW solutions were supplied by the research team of J.-P. Simonato from CEA-LITEN (Grenoble, France). They are synthesized following the protocol published by Mayousse et al.^30^ The nanowires had an average length of 8 μm and an average diameter of 70 nm. Alkaline earth boroaluminosilicate glass (Corning glass, Delta Technologies LTD) is used as the transparent substrate. For nanoscale transport measurements, thermally oxidized silicon wafers (200 nm thick SiO_2_ layer) are used. The sample sizes for electrical measurement and thermal stability tests are 12.5 × 12.5 mm^2^ and 25 × 25 mm^2^ for optical measurements. Before deposition, the substrates are sonicated in isopropanol for 10 min, rinsed with distilled water, and dried with nitrogen gas. AgNWs are deposited on these substrates by using a homemade spray-coater system (Figure S1). In this work, the pressure of the spray deposition is 1.4 bar and the temperature of the hot plate is 100 °C. The network density is assessed by image analysis of scanning electron microscopy (SEM), through the areal mass density (*amd*) expressed in mg/m^2^ of silver over the substrate.

For thermal annealing treatment, AgNW networks are heated at 200 °C for 1 h in air using a hot plate. For cold-welding treatment, the same spray-coater equipment used to fabricate AgNW networks is used. Type II water is sprayed with spray-coater equipment onto freshly deposited AgNW networks. The water flow rate is estimated at 1.2 ± 0.2 μL/s. The cold welding takes place during water evaporation. As for the deposition of AgNWs, the heating plate is set at 100 °C to quickly evaporate the water solvent, avoiding any coffee rings. The spray duration is short enough to clearly foresee such postdeposition treatment at an industrial scale. The spray was not static but moved continuously (at a maximum speed of 50 mm/s). Locally, the spray duration can be estimated to be around 5 s (see also Figure 1c,d).

**Figure 1:**
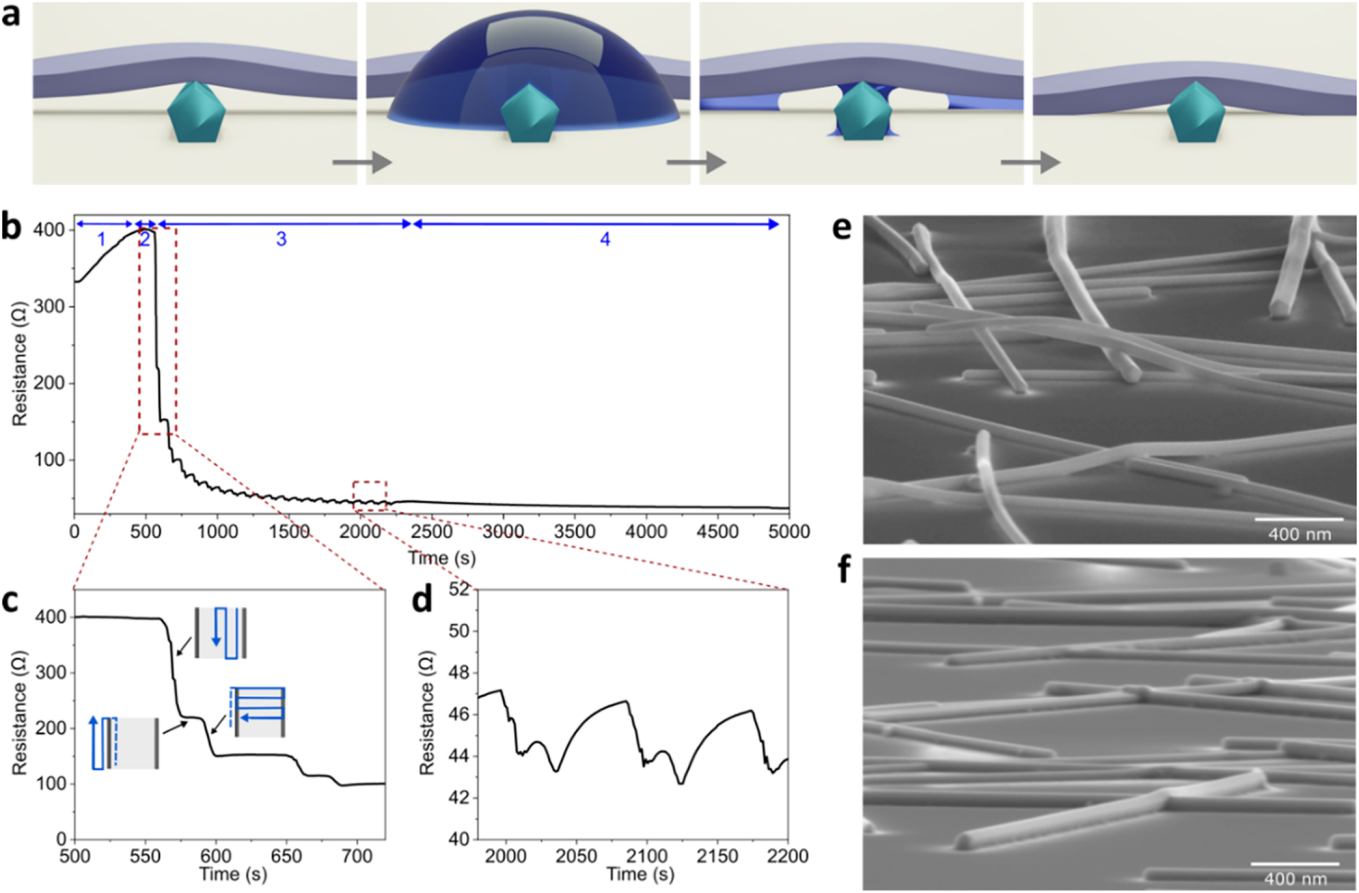
Principle of capillary-force-induced cold-welding treatment, in situ electrical resistance, and the effect on AgNW morphological properties. (a) Schematic principle of capillary-force-induced cold-welding between two nanowires, in purple and light green in the picture (Section 2.3.1). (b) Time dependence of the AgNW network resistance during capillary-force-induced cold-welding treatment. Blue arrows indicate the four domains described in the text. (c) Zoom of (b) on the first cold-welding cycle, the inset illustrates the water spraying scanning directions. (d) Zoom of (b) showing that after a few water spraying cycles, only a thermal reversible resistance change is observed. (e–f) Tilted Scanning electron microscopy (SEM) observations of (e) the AD-AgNW network and (f) the CW-AgNW network.

### II.II Structural and Optical Properties of AgNW Networks

The morphologies of AgNW networks are analyzed with a Field-Emission Gun Scanning Electron Microscopy (FEG-SEM, Zeiss Gemini 300, Carl Zeiss Microscopy GmbH) with an accelerating voltage of 3 keV. The areal mass density (*amd*), corresponding to the AgNW mass per unit of the surface, is estimated with the “Ridge detection” plugin of the ImageJ computer program (NIH, Bethesda). The detailed protocol to estimate *amd* values can be found in previous works.^31^ For each sample, seven SEM images are taken to calculate the *amd* averaged value. The *amd* of the three AgNW networks (AD-AgNWs, TA-AgNWs, and CW-AgNWs) is equal to 45 ± 6 mg/m^2^. For the nanoscale 4-point-probe (4PP) study, the *amd* of 12 ± 8 mg/m^2^ for the three networks is below the percolation threshold.^32^

The total transmittance as a function of the wavelength is measured with a PerkinElmer Lambda 950 UV–vis-NIR spectrophotometer (PerkinElmer) equipped with an integrating sphere. The wavelength range is from 250 to 2500 nm, and the step size is 10 nm.

### II. IIIElectrical Characterizations of AgNW Networks and NW–NW Junctions

#### II.III.I *In Situ* Macroscale Characterization

Silver paste (L-200N, CDS Electronique) contacts are deposited at two opposite ends of the studied AgNW networks and dried overnight in air. The thermal tests are performed in air: the samples are heated on a hot plate from room temperature to 400 °C with a heating ramp of 5 °C/min. The resistance is measured with a two-probe setup using a Keithley 2400 source measure unit (Keithley Instruments Inc.) while applying a voltage of 0.1 V (to avoid any degradation due to the Joule heating observed at larger applied voltage bias).^33^ The setup is controlled by LabVIEW software (National Instruments).

#### II.III.II Nanoscale Characterization

The electrical resistance at the scale of individual AgNW is measured using a Nanoprobe (Scienta-Omicron GmbH), composed of four independent Scanning Tunneling Microscopes (STM) under the supervision of an SEM in ultrahigh vacuum (UHV). The system is operated by a homemade controller (codeveloped by IEMN and Specs-Zurich GmbH), synchronized with Keithley 2636 source measure units (Keithley Instruments Inc.) for electrical four-probe measurements and supervised by homemade LabVIEW software (National Instruments).^34^ Such a combination allows a precise and gentle positioning of each tip independently at the nanoscale. The STM tungsten tips are chemically etched in KOH solution to obtain a sharp apex and annealed in UHV to remove the oxidation layer. The tips are brought into electrical contact on AgNWs with the help of the STM control system. The tips are maintained in contact with the feedback loop off, and the current can be monitored to keep it stable. The current induced by the SEM beam is several orders of magnitude lower than the current injected into the nanowires for electrical measurements. However, to avoid additional charging of the AgNW networks, 4-probe measurements are performed with the SEM turned off. The data analysis is performed with the Origin 2021b software.

## III Results and Discussion

### III.I Principle of Capillary-Force-Induced Cold-Welding and Electrical Observations at the Network Scale

The principle of capillary-force-induced cold welding is schematically depicted in Figure 1a at the scale of AgNW junctions. After the first step of spraying water, the evaporation process occurs. During this evaporation process, liquid water meniscus exists at the nanowire–nanowire junctions and at the nanowire–substrate junctions. The presence of these water menisci, which are associated with very small radius curvature (i.e., a few tens of nm), leads to strong capillary forces at both locations (AgNW junctions and AgNW-substrate interface). This results in local large pressures from tens of MPa up to GPa range,^16,19^ which is comparable to those associated with mechanical pressure treatments on AgNW networks.^14^ Zhang et al.^18^ investigated the effect of the substrate by estimating nanowire–substrate capillary force, which could be almost 10 times higher than nanowire–nanowire capillary force; the wire–substrate capillary force acts as a ≪zipper≫ and dominates the cold-welding process. Consequently, it implies on one side a strong contact between AgNWs, which decreases the electrical resistance of AgNW junctions and, subsequently, the electrical resistance of the whole network. On the other side, a drastic reduction of the roughness associated with the AgNW network is observed.

The *in situ* electrical resistance of a AgNW network measured during capillary-force-induced cold-welding is shown in Figure 1b. First, the resistance increases during the heating of the AgNW network up to 100 °C (domain 1), thanks to the metallic behavior of the AgNW network in the low-temperature range.^33^ The resistance is further stabilized with the AgNW temperature stabilization (domain 2). Then, a spraying series of water is performed onto the AgNW network (domain 3). As magnified in Figure 1c for each cold-welding cycle, a drop in resistance is observed, followed by a plateau of resistance and then another drop in resistance. The two decreases in resistance of one cycle occur because of water spraying along the *x*-direction and then the *y*-direction (inset of Figure 1c), which cools the AgNW network thanks to the temperature difference between water at room temperature and the AgNW network at 100 °C. The plateau is due to a delay between the spraying along the *x* and *y* directions (Figure S1). The final resistance value after one cycle is decreased compared to the one at the beginning of the cycle: a nonreversible resistance variation is indeed clearly observed. During a water spraying cycle, two physical mechanisms thus occur: a reversible thermal effect and a capillary effect, the latter being associated with both elastic and plastic deformation. After 1500 s, almost 10 water spraying cycles have been performed, and the electrical resistance is eventually around 43 Ω at 100 °C (while it was about 330 Ω before the cold-welding process). Thus, the contacts at NW–NW junctions have been optimized, and only a reversible thermal effect occurred (Figure 1d). Figure 1e,f shows SEM images, respectively, before and after cold welding with AgNWs, having more intimate contact at the junctions after the cold-welding process. In addition, the gap between the AgNWs and the substrate appears much smaller, and the rugosity of AgNW networks has been drastically reduced. These observations are in good agreement with previous works.^16,18^ As a result, the electrical resistance of the network after cold-welding treatment is 33 Ω at room temperature, leading to a resistance decrease of more than 90%. It has already been shown that the relative decrease of the resistance before and after post-treatment depends on the type and dimensions of AgNWs,^12^ complicating any meaningful comparison with other reported values. This motivates the present study on the comparison of AgNW networks from the same synthesis, but with different post-treatments (i.e., thermal annealing and cold-welding).

The electrical resistance values measured for AD-AgNW, TA-AgNW, and CW-AgNW networks are compared in Table 1. In this study, 20 AD-AgNW samples were measured, giving an average electrical resistance of 338 ± 23 Ω. Ten samples underwent thermal annealing or cold-welding treatments each. For the cold-welding postdeposition treatment, a series of 10 cycles of water spraying was performed to minimize the resistance of AgNW networks. As reported in Table 1, we find similar average electrical resistance values of 35 ± 2 and 36 ± 3 Ω for the TA-AgNWs and CW-AgNWs, respectively. The morphological changes are evaluated by comparing SEM images of AD, TA, and CW-AgNW networks (Figure 2a–c). In the case of conventional thermal annealing of AgNWs, a so-called “neck” formation is observed at NW–NW junction (Figure 2b). This stems from the minimization of the surface energy during the thermal sintering.^35^ In contrast, the cold-welded junctions do not exhibit any “neck” shape (Figure 2c), and the top nanowire instead appears nested in the bottom nanowire. Figure 2d depicts the total transmittance of AgNW networks before and after both postdeposition treatments. The total transmittances at 550 nm, after substrate subtraction, are reported in Table 1. The three curves are almost superposed on the whole wavelength range, showing a negligible effect of the postdeposition treatments on the optical transmittance, in agreement with a previous report.^18^

**Figure 2:**
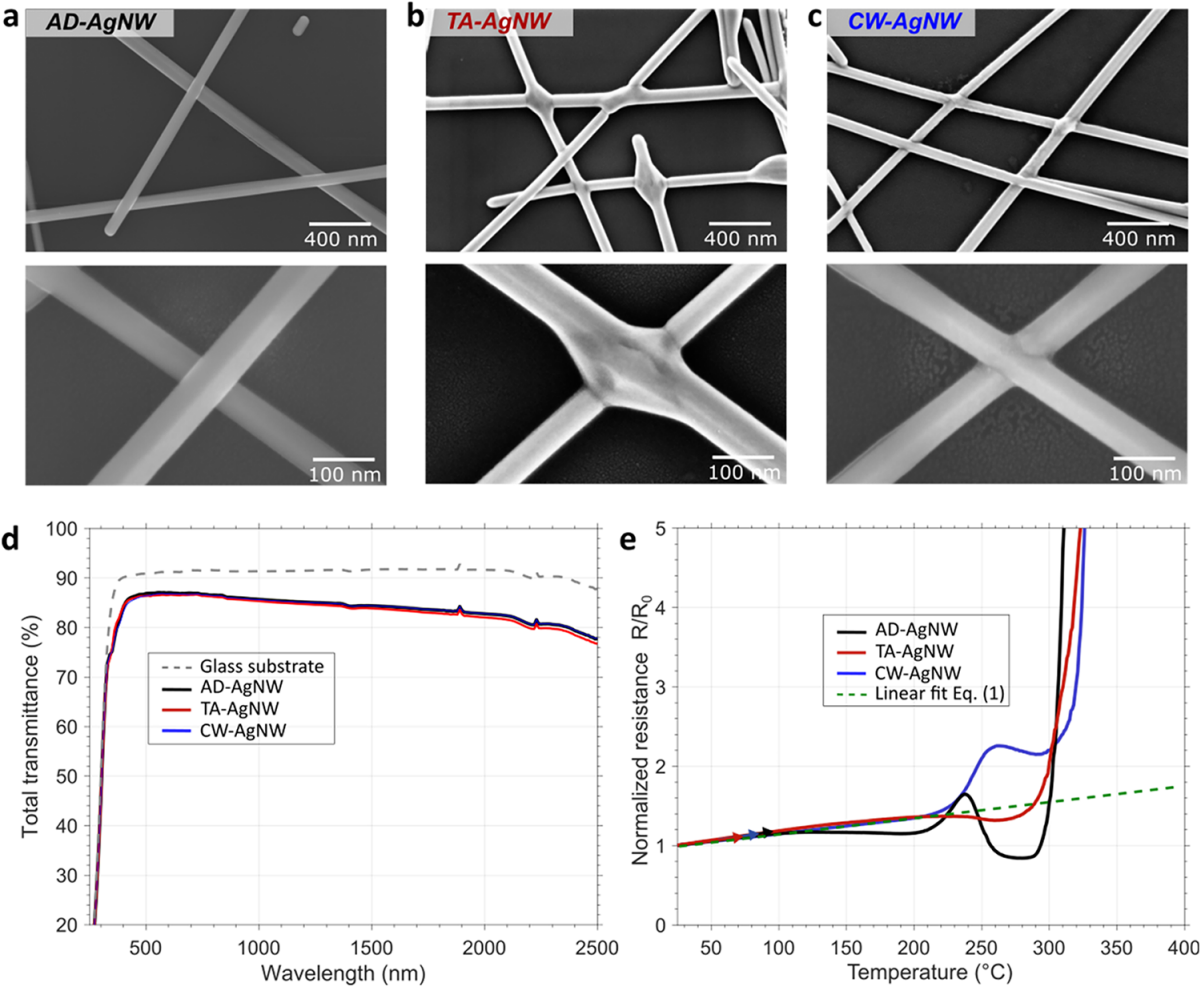
Effects of postdeposition treatments on the morphological, optical, and thermal stability properties of AgNW networks. (a–c) SEM images of AD, TA, and CW-AgNW networks. The bottom images are a magnification of the top images. (d) Total transmittance for the three types of networks. The transmittance of the substrate is also shown for comparison. (e) Normalized electrical resistance as a function of temperature for the three types of AgNW networks during the thermal ramp in air, with a temperature rate of 5 °C/min. The green dashed line indicates the fit with eq 1.

**Table 1:** Electrical Resistance *R* and Total Transmittance *T*_r_, of AD, TA, and CW-AgNW Networks^a^

	parameters of postdeposition treatments	*R* (Ω)	*T*_r_ (%)
As-deposited (AD) AgNWs	-	338 ± 23	94.3
Thermally annealed (TA) AgNWs	200 °C/1 h	35 ± 2	94.1
Cold-welded (CW) AgNWs	100 °C/1.2 ± 0.2 μL/s/10 water spraying cycles (<15 min)	36 ± 3	94.0

a *R* is an average value over 10 samples. *T*_r_ values have been obtained at 550 nm after subtraction of the glass substrate contribution.

It is of prime importance to assess the thermal stability of AgNW networks, as it could affect the reliability of the entire device when integrated. The response to the thermal ramp in air of AD-AgNW, TA-AgNW, and CW-AgNW is reported in Figure 2e. First, the resistance increases linearly with temperature for temperatures up to 160 °C for AD-AgNW and up to 200 °C for TA-AgNW and CW-AgNW. This corresponds to the expected linear thermal dependence of the resistance in metals, and can be described as follows^36^

1 $$R\left(T_{0}+\Delta T\right)=R\left(T_{0}\right)\times \left(1+\beta \times \Delta T\right)$$

where *T*_0_ is the room temperature (*RT*), assumed to be 20 °C in this study, Δ*T* is the temperature increase, *R*_0_(*T*_0_) is the AgNW network resistance at *RT*, and β is the temperature coefficient of resistivity. The fit with eq 1 (green dashed line in Figure 2e) gives β = 2.3 × 10^–3^ K^–1^ in agreement with a previous estimation on the AgNW network.^6^

Next, the AD-AgNW networks exhibit a small decrease in the normalized resistance (at about 260–280 °C), which is due to sintering effects associated with AgNW of small diameters. This decrease is not observed for TA-AgNW and CW-AgNW since the junctions have already been optimized during these postdeposition treatments. Then, a hump can be observed around 230 °C for the AD-AgNW and CW-AgNW. This is due to the degradation of some AgNWs and associated junctions of smaller AgNW diameters within the network. Indeed, the smaller the AgNW diameters, the lower the degradation temperature.^10,37^ This hump is not observed for the thermally annealed AgNWs since the degradation of small junctions already occurred during their optimization by thermal annealing.^38^ The normalized resistance of AD-AgNW significantly decreases below the linear fitting, around 270 °C. This decrease is due to the thermal sintering of the majority of AgNW junctions.^6^ For CW-AgNW, this phenomenon is not observed, since AgNW junctions were already fully optimized. Thus, it is not necessary to perform conventional thermal annealing or any other postdeposition treatment after cold-welding treatment. Between 240 and 290 °C, one can observe that the resistance increase for the CW-AgNW is significantly higher than that for the AD-AgNWs or TA-AgNWs. The origin of such observation is not trivial, since, as shown by Figure 2a–c, the junction morphologies for these 3 types of AgNW networks appear very different. A deep understanding of such observation would require detailed structural analysis of the junctions, which is out of the scope of this article. CW-AgNW networks have never been subjected to temperatures above 100 °C before the thermal ramp shown in Figure 2e, while the TA-AgNW network (annealed at 200 °C for 1 h) had time to reduce structural defects within the junctions. The thermal ramp rate (5 °C/min) is too fast to play such a role for CW-AgNW. In addition to the highly probable presence of defects in CW-AgNW, the dimensions of the junction are much smaller (see Figure 2c) compared to those for TA-AgNW (see Figure 2b).

An additional interesting point concerns the comparison of the electrical resistance between CW-AgNW and TA-AgNW. Although the optimization mechanisms appear different, the electrical resistance appears similar (Table 1). This apparent contradiction can be explained by the fact that an optimization process does not have the same effects depending on the AgNW diameter. For instance, TA-AgNW exhibits probably optimized junctions for intermediate AgNW diameters (but smaller junctions have been degraded during the thermal annealing), while CW-AgNW exhibits probably optimized junctions for smaller AgNW diameters (but larger junctions have not been optimized since Laplace capillary pressure decreases inversely with curvature radius). The electrical resistance after these two treatments appears similar (see Table 1), but the latter have very probably optimized junctions of different sizes. Since smaller dimensions undergo morphological instability at lower temperature, this contributes as well to the higher resistance increase for CW-AgNW compared to TA-AgNW in the temperature range of 240–290 °C. Finally, for temperatures larger than 290 °C, the electrical resistance increases drastically for the three types of AgNW networks due to morphological instability associated with AgNWs spheroidization (i.e., Plateau-Rayleigh instability).,^6,36,39^ This result demonstrates that the cold-welding process does not worsen the thermal stability of AgNW networks up to 290 °C.

### III.II Electrical Characterization at the Scale of NW–NW Junctions

The electrical resistance has been measured at the scale of individual AgNW in sparse AD, TA, and CW-AgNW networks using nanoscale 4PP. Figure 3 presents typical results: Figure 3a,b shows SEM images of a typical probe configuration used to determine the resistivity ρ_AgNW_ of an individual AgNW and the junction resistance *R*_J_ between two AgNWs in contact with each other. The resistance is measured in a 4-probe geometry that eliminates the contribution of the contact resistance between the probes and the AgNWs.^40^ The two outer tips (in purple in Figure 3a,b) are used to inject the current, and the probed region corresponds to the region between the two inner tips (in blue in Figure 3a,b), where the voltage drop is measured. As the silicon substrate is covered by an insulating oxide layer, the current only flows through the AgNWs. In Figure 3a, the inner tips are placed along the same AgNW and allow probing of the electrical resistivity of an individual nanowire (here NW1). In Figure 3b, the inner tips are placed on either side of an NW–NW junction to probe *R*_J_.

**Figure 3:**
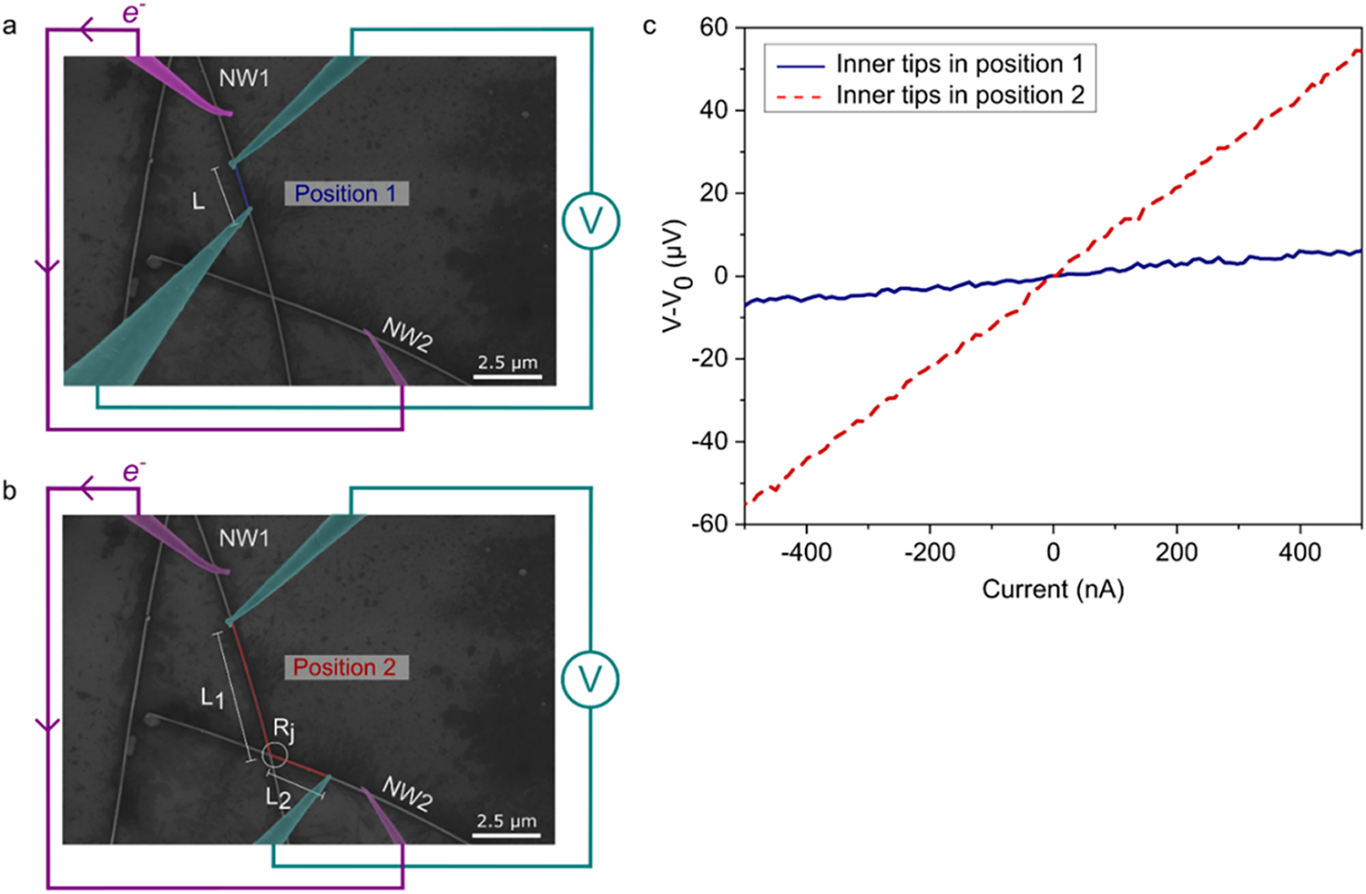
Electrical resistivity of individual AgNW and junction resistance measurements by four-probe measurements at the nanoscale. (a, b) SEM images of a junction between two AgNWs (NW1 and NW2) and 4 tips. (a) The inner tips (colored in blue) are on the same AgNW (NW1) to determine its electrical resistivity. (b) The inner tips surround a junction between two AgNWs to probe the junction resistance, *R*_J_. The electrical circuit is superimposed: the outer tips (colored in purple) are connected to the current source, and the inner tips are connected to the voltmeter. The probed region is colored in blue and red in (a) and (b), respectively. (c) Corresponding measured *I*–*V* characteristics associated with position 1 (a) and position 2 (b).

The current–voltage characteristics measured in both cases, shown in Figure 3c, exhibit a linear behavior, as expected for an ohmic resistance. An instrumental offset of *V*_0_ = 62 μV has been subtracted in the graph for clarity. The slope of the *I*–*V* curve gives the electrical resistance *R* between the two inner tips. In the first configuration, ρ_AgNW_ is directly obtained according to the Pouillet law:

2 $$\rho_{\mathrm{AgNW}}=\frac{RS_{\mathrm{AgNW}}}{L}$$

with *S*_AgNW_ being the section of the AgNW under study and *L* is the distance between the inner tips, both dimensions being extracted from the SEM images of each nanowire. In the second configuration, *R* is equal to the sum of *R*_J_ and the resistance of a segment of NW1 (*R*_1_) and NW2 (*R*_2_). Thus, the junction resistance *R*_J_ is determined as follows

3 $$R_{J}=R-\left(R_{1}+R_{2}\right)$$

*R*_1_ and *R*_2_ are calculated using eq 2 with *L*_1_ and *L*_2_ measured by SEM.

Probed AgNW and junctions were chosen randomly, without applying geometrical selection criteria, so that the data set reflects the diversity of experimentally accessible configurations. The geometries of all measured junctions are provided in Figure S3. All of the measurements presented were obtained with maximum currents below 500 nA, to avoid possible sintering of the NW–NW junctions by the Joule effect.^27^ In our study, we have observed a systematic ohmic behavior, including for AD-AgNW junctions. In addition, a dense AgNW network has been studied at a larger scale with the Nanoprobe (Figure S2) to evaluate the connexion between the properties of the one-dimensional single AgNW and the two-dimensional AgNW network: as expected, the resistance is increasing linearly with distance along one nanowire while the resistance of the whole AgNW network does not vary with the distance.^39^

The electrical resistivity and junction resistance values are summarized in Figure 4a,b for the three types of AgNW networks. For each type, 10 nanowires have been probed to determine ρ_AgNW_ and 10 junctions have been measured to determine *R*_J_. As shown in Figure 4a, the measured resistivities are dispersed between 15 and 36 Ω•nm, with similar values for AD-AgNWs, TA-AgNWs, and CW-AgNWs. The average resistivity of 26 ± 5 Ω.nm for the three samples is in good agreement with previous reports for AD and TA-AgNWs.^27,29,41^ The similar results obtained for the three AgNW networks confirm that no change of resistivity was induced by both methods of postdeposition treatment and very probably point to an efficient capping by the PVP layer against oxidation. For comparison, Figure 4a also shows the silver bulk resistivity^42^ ρ_Agbulk_ equal to 16 Ω.nm (blue solid line). When the electron mean free path Λ is of the order of the NW diameter, the resistivity of a NW is larger than the bulk resistivity due to the electron scattering at the NW surface.^43^ The resistivity of a single AgNW is given by^39^

4 $$\rho =\rho_{\mathrm{Agbulk}}\left(1+\frac{\Lambda }{2D_{\mathrm{AgNW}}}\right)$$

with *D*_AgNW_ being the AgNW diameter. The resistivity obtained with eq 4, assuming Λ ≃ 50 nm^44^ (black dotted line in Figure 4a), gives a relatively stable resistivity 21 ± 1 Ω·nm in the relevant AgNW diameter range. The slightly higher measured resistivities are probably due to increased electron scattering caused by defects on the AgNWs surface.^40^ The electrical resistivity of individual metallic nanowires has been extensively investigated, particularly in the case of noble metals such as Ag and Cu.^41^ When a metallic structure is confined to nanometric dimensions comparable to the electronic mean free path along one direction, resistivity is enhanced as a consequence of size-dependent effects.^41^ This increase arises from various electron-scattering mechanisms associated with the high surface-to-volume ratio, including surface scattering, impurities, and structural imperfections (e.g., multiple twin boundaries or surface roughness), with the latter generally exerting the strongest influence.^45^ Furthermore, local variations in resistivity may originate from heterogeneities in nanowire diameter as well as from spatially nonuniform defects, such as impurities or stacking faults. According to Matthiessen’s rule, the contributions of these scattering processes add linearly to the overall resistivity. As a result, the experimentally measured resistivity of Ag nanowires typically exceeds the theoretical predictions and exhibits significant variability due to the presence and spatial distribution of such defects.

**Figure 4:**
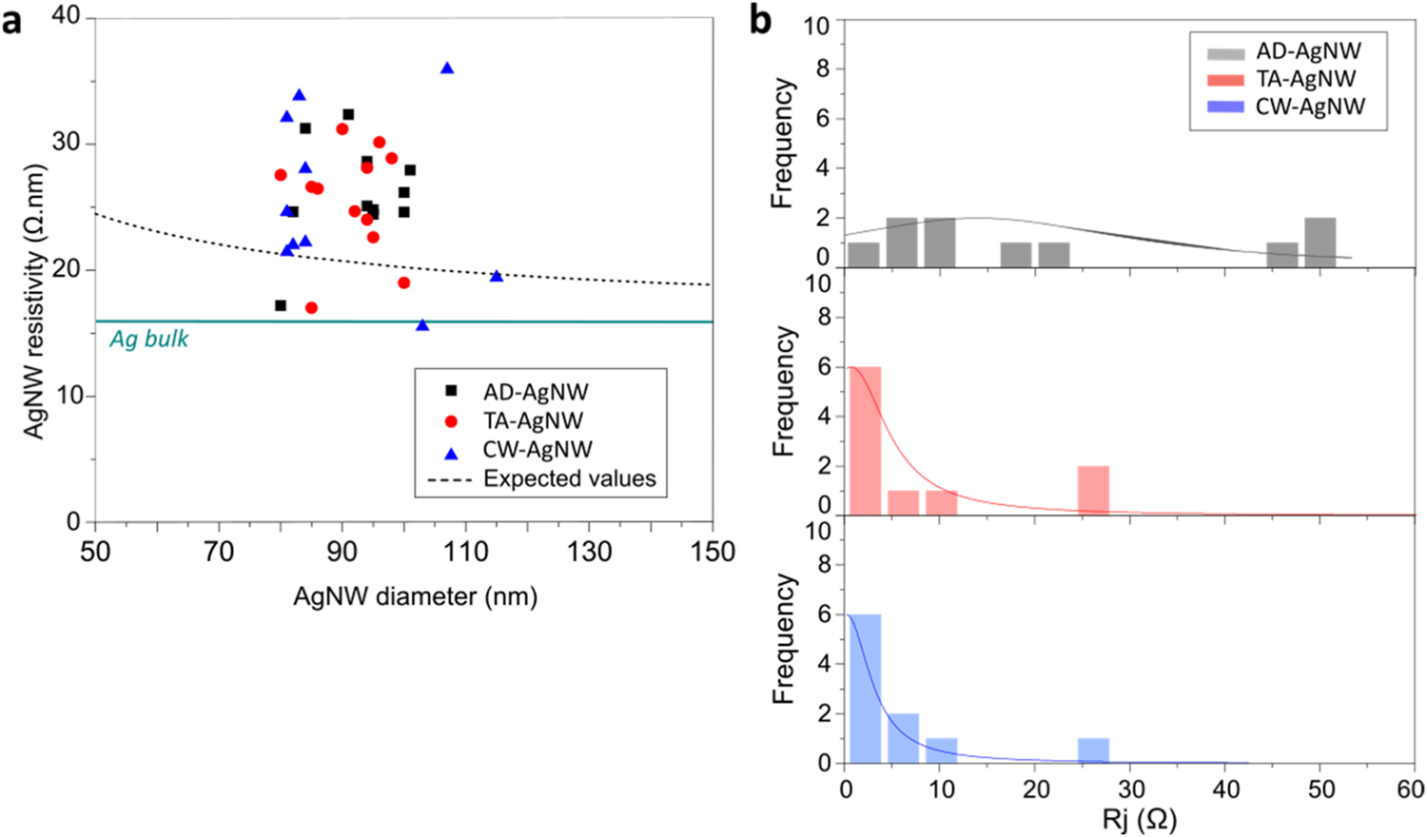
Effect of postdeposition on electrical resistivity and NW–NW junction resistances. (a) Electrical resistivity as a function of AgNW diameter for AD-AgNWs, TA-AgNWs, and CW-AgNWs. The black dashed line shows the fit of the whole data set with eq 4. (b) Distribution of the junction resistances for AD-AgNWs, TA-AgNWs, and CW-AgNWs. For each sample, experimental data have been fitted by a Lorentzian (continuous line).

The measured values of the NW–NW junction resistance for AD-AgNWs, TA-AgNWs, and CW-AgNWs are presented in Figure 4b. All probed NW–NW junctions were also examined by SEM, as shown in Figure S3. The average junction resistance decreases significantly with both postdeposition treatments, dropping from *R*_J_^AD^ = 21 ± 6 Ω for AD-AgNWs to *R*_J_^TA^ = 6 ± 3 Ω for TA-AgNWs and *R*_J_^CW^ = 5 ± 3 Ω for CW-AgNWs. Furthermore, the dispersions of the *R*_J_ values differ noticeably, as highlighted by the Lorentzian fit shown in the figure. While *R*_J_^AD^ exhibits a relatively broad dispersion from 0 to 48 Ω, 60% of *R*_J_^TA^ and *R*_J_^CW^ values fall below 1 Ω, consistent with expectations for optimized junctions. We attribute the 20% of the *R*_J_^TA^ and *R*_J_^CW^ values in the 1–12 Ω to the experimental uncertainty on the calculated junction resistance due to the uncertainties on nanowire size, silver resistivity, and measured resistance. In all three cases, a small second peak is present at higher resistance. This peak is located at 47 Ω for AD-AgNW and shifts to 26 and 27 Ω for the two postdeposition methods, suggesting a second minority population of junctions of a different type. However, SEM images (Figure S3) did not reveal any variation in morphology with junction resistance. These higher resistances may thus stem from local network granularity between the AgNWs or junction contamination. The exhaustive structure and composition of each junction are out of the scope of this study and require a dedicated investigation.

The NW–NW junction resistance has been reported by a few authors in the case of as-deposited and thermally annealed AgNWs.^27,29^ As shown by Bellew et al.,^27^ an optimal thermal annealing is at the origin of a large decrease in the junction resistance. This result is in good agreement with our observations for TA-AgNWs, and it confirms the efficient welding of TA-AgNWs at the junction. Additionally, our work shows that a very analogous result is obtained for CW-AgNWs, with most of the junctions having resistances below our experimental sensitivity. These results are compatible with the scenario proposed by Liu et al.,^16^ where the presence of moisture induces the flowing and reprecipitation of the PVP layer and proves that capillary-force-induced cold-welding is as efficient as conventional thermal annealing to optimize the NW–NW junctions, however, working at a lower temperature.

## IV Conclusion

In this study, the capillary-force-induced cold-welding has been explored as an alternative to conventional thermal annealing for postdeposition treatment. This technique involves spraying water onto AgNW networks at relatively low temperatures (around 100 °C). By monitoring the electrical resistance of AgNW networks *in situ* during cold-welding, it has been demonstrated that only 10 cycles of water spraying (approximately 15 min) are required to achieve optimal electrical resistance in these networks. SEM imaging further revealed enhanced junction contact between AgNWs and improved surface smoothness, consistent with findings from previous studies. These improvements hold promise for reducing short-circuit risks in applications such as organic solar cells.

In parallel, the effects of cold-welding on the morphology, optical transmittance, electrical resistance, and thermal stability of AgNW networks were examined, comparing these results with those from traditional thermal annealing (200 °C for 1 h). Despite some morphological differences at the AgNW junctions, both treatments achieved comparable reductions in electrical resistance without significant changes in optical transmittance. Electrical resistance was also measured at the scale of individual AgNWs, specifically assessing NW–NW junction resistance with a nanoscale 4PP resistance measurement. For the as-deposited AgNWs, the resistances were smoothly dispersed between 0 and 51 Ω. After thermal annealing or cold-welding, the average values were nearly identical, with 60% of the junctions being less than 1 Ω in both cases. These results confirm that both postdeposition treatments are equally effective in reducing electrical resistance at both nanoscale and macroscale levels.

These findings highlight the strong potential of capillary-force-induced cold-welding as a viable method for optimizing the electrical resistance in other metallic nanowire networks. This method could also align well with roll-to-roll technology, enabling the scalable production of flexible transparent electrodes using metallic nanowire networks.

## Supplementary Material



## Notes

Details on the sample preparation and the nanoscale electrical measurements (PDF)

## References

[1] Inganäs O. (2011). Avoiding indium. Nat. Photonics.

[2] Hu L., Kim H. S., Lee J.-Y., Peumans P., Cui Y. (2010). Scalable Coating
and Properties of Transparent, Flexible, Silver Nanowire Electrodes. ACS Nano.

[3] Hu H., Wang S., Wang S., Liu G., Cao T., Long Y. (2019). Aligned Silver Nanowires Enabled Highly Stretchable and Transparent
Electrodes with Unusual Conductive Property. Adv. Funct. Mater..

[4] Lee S. J., Kim Y.-H., Kyu Kim J., Baik H., Hoon Park J., Lee J., Nam J., Hyeok Park J., Lee T.-W., Yi G.-R., Ho Cho J. (2014). A roll-to-roll
welding process for planarized silver nanowire electrodes. Nanoscale.

[5] Jung E., Kim C., Kim M., Chae H., Cho J. H., Cho S. M. (2017). Roll-to-roll preparation
of silver-nanowire transparent electrode and its application to large-area
organic light-emitting diodes. Org. Electron..

[6] Langley D. P., Lagrange M., Giusti G., Jiménez C., Bréchet Y., Nguyen N. D., Bellet D. (2014). Metallic nanowire
networks: effects of thermal annealing on electrical resistance. Nanoscale.

[7] Yang Z., Guo Y., Guo W., Zhao M., Wang H., Wei B., Miao Y., Guo K. (2025). MXene/AgNWs/MXene Sandwich-Structured
Transparent Electrode for High-Performance Flexible OLEDs. Small.

[8] Ding Y., Cui Y., Liu X., Liu G., Shan F. (2020). Welded silver nanowire networks as high-performance
transparent conductive electrodes: Welding techniques and device applications. Appl. Mater. Today.

[9] Nguyen V.-T., Phan G. A. V. (2023). Atomistic insight into welding silver
nanowires and interfacial characteristics of the welded zone. Mater. Today Commun..

[10] Lagrange M., P Langley D., Giusti G., Jiménez C., Bréchet Y., Bellet D. (2015). Optimization of silver nanowire-based
transparent electrodes: effects of density, size and thermal annealing. Nanoscale.

[11] Teymouri A., Pillai S., Ouyang Z., Hao X., Liu F., Yan C., Green M. A. (2017). Low-Temperature
Solution Processed Random Silver Nanowire as a Promising Replacement
for Indium Tin Oxide. ACS Appl. Mater. Interfaces.

[12] Bardet L., Papanastasiou D. T., Crivello C., Akbari M., Resende J., Sekkat A., Sanchez-Velasquez C., Rapenne L., Jiménez C., Muñoz-Rojas D., Denneulin A., Bellet D. (2021). Silver Nanowire Networks:
Ways to Enhance Their Physical Properties and Stability. Nanomaterials.

[13] Du M., Yang Z., Miao Y., Wang C., Dong P., Wang H., Guo K. (2024). Facile Nanowelding Process for Silver
Nanowire Electrodes Toward High-Performance Large-Area Flexible Organic
Light-Emitting Diodes. Adv. Funct. Mater..

[14] Tokuno T., Nogi M., Karakawa M., Jiu J., Nge T. T., Aso Y., Suganuma K. (2011). Fabrication of silver
nanowire transparent electrodes at room temperature. Nano Res..

[15] Tseng J.-Y., Lee L., Huang Y.-C., Chang J.-H., Su T.-Y., Shih Y.-C., Lin H.-W., Chueh Y.-L. (2018). Pressure
Welding of Silver Nanowires Networks at Room Temperature as Transparent
Electrodes for Efficient Organic Light-Emitting Diodes. Small.

[16] Liu Y., Zhang J., Gao H., Wang Y., Liu Q., Huang S., Guo C. F., Ren Z. (2017). Capillary-Force-Induced Cold Welding in Silver-Nanowire-Based Flexible
Transparent Electrodes. Nano Lett..

[17] Weiß N., Müller-Meskamp L., Selzer F., Bormann L., Eychmüller A., Leo K., Gaponik N. (2015). Humidity assisted annealing technique for transparent
conductive silver nanowire networks. RSC Adv..

[18] Zhang K., Li J., Fang Y., Luo B., Zhang Y., Li Y., Zhou J., Hu B. (2018). Unraveling
the solvent induced welding of silver nanowires for high performance
flexible transparent electrodes. Nanoscale.

[19] Lu Y., Huang J. Y., Wang C., Sun S., Lou J. (2010). Cold welding of ultrathin gold nanowires. Nat. Nanotechnol..

[20] Xu F., Xu W., Mao B., Shen W., Yu Y., Tan R., Song W. (2018). Preparation and cold welding of silver nanowire based transparent
electrodes with optical transmittances > 90% and sheet resistances
< 10 ohm/sq. J. Colloid Interface Sci..

[21] Lu H., Zhang D., Cheng J., Liu J., Mao J., Choy W. C. H. (2015). Locally Welded Silver Nano-Network
Transparent Electrodes with High Operational Stability by a Simple
Alcohol-Based Chemical Approach. Adv. Funct.
Mater..

[22] Kang H., Kim Y., Cheon S., Yi G.-R., Cho J. H. (2017). Halide Welding for
Silver Nanowire Network Electrode. ACS Appl.
Mater. Interfaces.

[23] Madeira A., Papanastasiou D. T., Toupance T., Servant L., Tréguer-Delapierre M., Bellet D., Goldthorpe I. A. (2020). Rapid synthesis of ultra-long silver
nanowires for high performance transparent electrodes. Nanoscale Adv..

[24] Betker M., Harder C., Erbes E., Heger J. E., Alexakis A. E., Sochor B., Chen Q., Schwartzkopf M., Körstgens V., Müller-Buschbaum P., Schneider K., Techert S. A., Söderberg L. D., Roth S. V. (2023). Sprayed Hybrid Cellulose
Nanofibril–Silver Nanowire Transparent Electrodes for Organic
Electronic Applications. ACS Appl. Nano Mater..

[25] Gabbett C., Kelly A. G., Coleman E., Doolan L., Carey T., Synnatschke K., Liu S., Dawson A., O’Suilleabhain D., Munuera J., Caffrey E., Boland J. B., Sofer Z., Ghosh G., Kinge S., Siebbeles L. D. A., Yadav N., Vij J. K., Aslam M. A., Matkovic A., Coleman J. N. (2024). Understanding how
junction resistances impact the conduction mechanism in nano-networks. Nat. Commun..

[26] Fata N., Mishra S., Xue Y., Wang Y., Hicks J., Ural A. (2020). Effect of junction-to-nanowire
resistance ratio on the percolation conductivity and critical exponents
of nanowire networks. J. Appl. Phys..

[27] Bellew A. T., Manning H. G., Gomes da Rocha C., Ferreira M. S., Boland J. J. (2015). Resistance
of Single Ag Nanowire Junctions and Their Role in the Conductivity
of Nanowire Networks. ACS Nano.

[28] Manning H. G., Flowers P. F., Cruz M. A., Rocha C. G. da., Callaghan C. O., Ferreira M. S., Wiley B. J., Boland J. J. (2020). The Resistance of Cu Nanowire–Nanowire Junctions
and Electro-Optical Modeling of Cu Nanowire Networks. Appl. Phys. Lett..

[29] Selzer F., Floresca C., Kneppe D., Bormann L., Sachse C., Weiß N., Eychmüller A., Amassian A., Müller-Meskamp L., Leo K. (2016). Electrical limit of silver nanowire electrodes: Direct measurement
of the nanowire junction resistance. Appl. Phys.
Lett..

[30] Mayousse C., Celle C., Moreau E., Mainguet J.-F., Carella A., Simonato J.-P. (2013). Improvements in purification of silver nanowires by
decantation and fabrication of flexible transparent electrodes. Application
to capacitive touch sensors. Nanotechnology.

[31] Nguyen V. H., Resende J., Papanastasiou D. T. (2019). Low-cost fabrication of flexible transparent electrodes based on
Al doped ZnO and silver nanowire nanocomposites: impact of the network
density. Nanoscale.

[32] Langley D. P., Lagrange M., Nguyen N. D. (2018). Percolation in networks of 1-dimensional objects: Comparison between
Monte Carlo simulations and experimental observations. Nanoscale Horiz..

[33] Bardet L., Akbari M., Crivello C., Rapenne L., Weber M., Nguyen V. H., Jiménez C., Muñoz-Rojas D., Denneulin A., Bellet D. (2023). SnO2-Coated Silver
Nanowire Networks as a Physical Model Describing Their Reversible
Domain under Electrical Stress for Stable Transparent Electrode Applications. ACS Appl. Nano Mater..

[34] Berthe, M. Combined STM and Four-Probe Resistivity Measurements on Single Semiconductor Nanowires. In Atomic Scale Interconnection Machines. Advances in Atom and Single Molecule Machines; Joachim, C. , Ed.; Springer: Berlin, Heidelberg, 2012.

[35] Xue S., Garlyyev B., Auer A., Kunze-Liebhäuser J., Bandarenka A. S. (2020). How the Nature of the Alkali Metal Cations Influences
the Double-Layer Capacitance of Cu, Au, and Pt Single-Crystal Electrodes. J. Phys. Chem. C.

[36] Raj KA S., Mane P., Radhakrishnan S., Chakraborty B., Rout C. S. (2022). Heterostructured Metallic 1T-VSe2/Ti3C2Tx
MXene Nanosheets for Energy Storage. ACS Appl.
Nano Mater..

[37] Patil J. J., Chae W. H., Trebach A., Carter K.-J., Lee E., Sannicolo T., Grossman J. C. (2021). Failing Forward: Stability of Transparent
Electrodes Based on Metal Nanowire Networks. Adv. Mater..

[38] Bardet L., Roussel H., Saroglia S., Akbari M., Muñoz-Rojas D., Jiménez C., Denneulin A., Bellet D. (2024). Exploring the Degradation of Silver
Nanowire Networks under Thermal Stress by Coupling *In Situ* X-Ray Diffraction and Electrical Resistance Measurements. Nanoscale.

[39] Vigonski S., Jansson V., Vlassov S., Polyakov B., Baibuz E., Oras S., Aabloo A., Djurabekova F., Zadin V. (2018). Au nanowire junction breakup through surface atom diffusion. Nanotechnology.

[40] Miccoli I., Edler F., Pfnür H., Tegenkamp C. (2015). The 100th anniversary of the four-point probe technique:
the role of probe geometries in isotropic and anisotropic systems. J. Phys.: Condens. Matter.

[41] Bid A., Bora A., Raychaudhuri A. K. (2006). Temperature
dependence of the resistance of metallic nanowires of diameter ⩾15nm:
Applicability of Bloch-Grüneisen theorem. Phys. Rev. B.

[42] CRC Handbook of Chemistry and Physics, 102nd ed.; Rumble, J. R. , Ed.; CRC Press: Boca Raton London New York, 2021.

[43] Huang Q., Lilley C. M., Bode M. (2009). Surface scattering
effect on the electrical resistivity of single crystalline silver
nanowires self-assembled on vicinal Si (001). Appl. Phys. Lett..

[44] Gall D. (2016). Electron mean free path in elemental
metals. J. Appl. Phys..

[45] Chawla J. S., Gstrein F., O’Brien K. P., Clarke J. S., Gall D. (2011). Electron scattering
at surfaces and grain boundaries in Cu thin films and wires. Phys. Rev. B.

