# Supplementary material for: In Situ Multiscale Investigation of Capillary-Force-Induced Cold-Welding of Silver Nanowire Networks
Source: ACS Omega. 2025 Nov 14;10(46):55716–24. doi: 10.1021/acsomega.5c07063 (PMC12658620; doi:10.1021/acsomega.5c07063)
Supplement: Supplementary file 1 [file ao5c07063_si_001.pdf]

Supplementary Materials

In situ multiscale investigation of capillary force-induced cold-welding of silver nanowire networks

Yevheniia Chernukha,[#1] Laetitia Bardet,[#2] Maxime Berthe,[1] Thomas Lerond,[1] Jean-Paul Mazellier,[3] Laurent Gangloff,[3] Aurore Denneulin,[2] Pascale Diener,[*1] and Daniel Bellet[2]

[1)] *Univ. Lille, CNRS, Centrale Lille, Univ. Polytechnique Hauts-de-France, Junia-ISEN, UMR 8520 - IEMN, F-59000 Lille, France*

[2)] *Univ. Grenoble Alpes, CNRS, Grenoble INP, LMGP, 38000 Grenoble, France*

[3)] *Thales Research and Technology, Route Départementale 128, 91767 Palaiseau Cedex, France*

[#)] *These authors contributed equally to this work*

[*)] *Corresponding author*

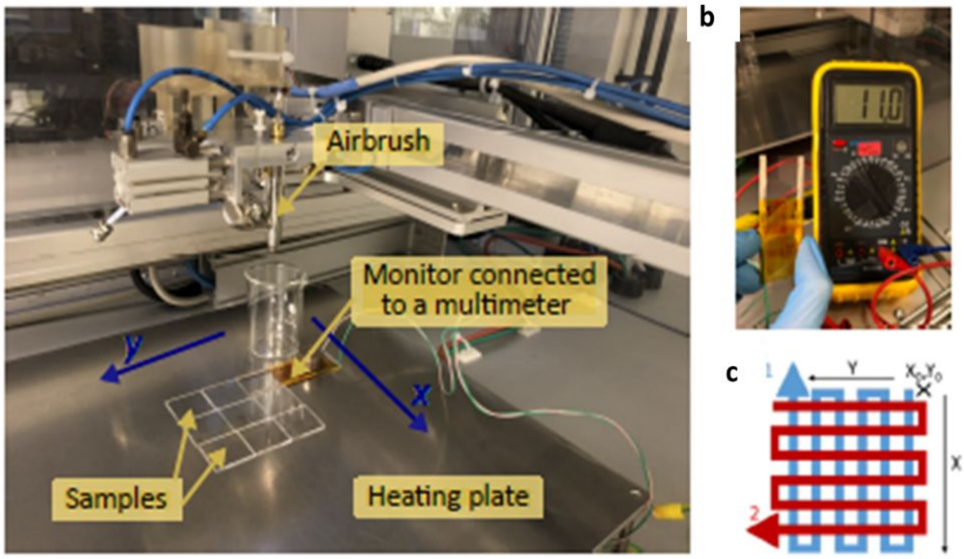

FIG. S1. Spray-coating setup used for the fabrication of AgNW networks. (a) Photo of the spray-coating setup used at LMGP. (b) Photo of the monitor used to measure *in situ* electrical resistance during deposition. The monitor is connected to an external multimeter. (c) During deposition, the spray-gun is moving along a regular and periodic array in x- and y- directions above an aluminum plate. One spraying cycle is completed after a "zigzag" movement along the x-direction and then along the y direction. The blue and red arrows represent the "zigzag" movement of the airbrush during one spraying cycle.

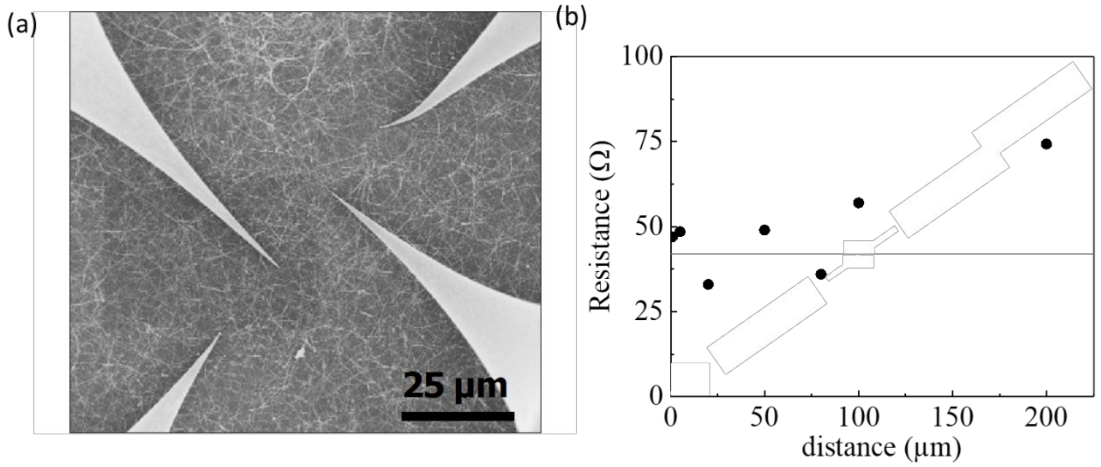

FIG. S2. Four-point probes transport measurements of a dense AgNW network. (a) SEM picture of the network, with the four tips that can be positioned at the desired interelectrode distance by high precision piezoelectric elements**.** (b) Corresponding measured resistance, for several interelectrode distances (black points). A constant resistance with distance, as expected for purely two-dimensional transport, is illustrated by the continuous grey line.

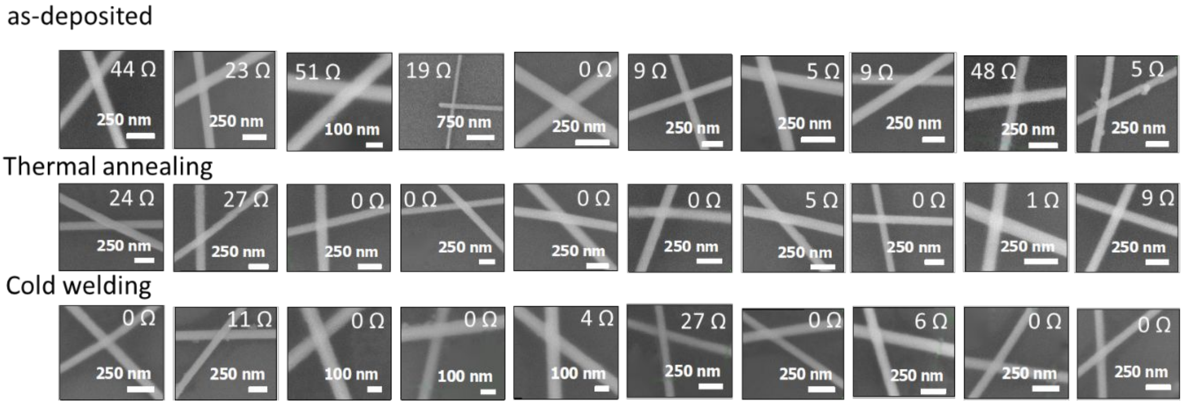

FIG. S3. SEM pictures of the NW-NW junctions probed by 4PP transport measurements. Each line corresponds to one AgNW network, indicated above: AD-AgNW, TA-AgNW, CW-AgNW. The measured values of the junction resistances are indicated for each junction.
